# Supplementary material for: Foster children’s perspectives on participation in child welfare processes: A meta-synthesis of qualitative studies
Source: PLoS One. 2022 Oct 10;17(10):e0275784. doi: 10.1371/journal.pone.0275784 (PMC9550086; doi:10.1371/journal.pone.0275784)
Supplement: S3 Table — (DOCX) [file pone.0275784.s003.docx]

**S3 Table – Participant and Study Characteristics**

| **Study ID, Country** | **Study Purpose** | **Qualitative methods** | **N (child)** | **Child Age, Gender, and Race or Ethnicity** | **% Care Type, Time in Care, Number of placements** |
| --- | --- | --- | --- | --- | --- |
| Degener 2020  The Netherlands | Addresses the ethnic identity of transculturally placed adolescent foster youth with ethnic minority backgrounds in The Netherlands | Interviews, photo elicitation methods | 20 | ***Age:*** 11-19  ***Gender or sex:*** 10 girls, 10 boys  ***Race/ethnicity:*** Moroccan and Dutch (1), Turkish and Dutch (1), Caribbean and Dutch (1),  Surinamese and Turkish (1), Moroccan (4), Surinamese (1), Caribbean (5), East-  African (5), Brazilian (1) | ***Care type:*** Foster care  ***Time in care:*** 2 weeks-12 years  ***Time in current placement:*** At least 6 months  ***Number of placements:***  Not stated |
| Wissö 2019  Sweden | Highlights that who counts as family and as a parent is ambiguous | Interviews | 11 | ***Age:*** 14-19  ***Gender or sex:*** 6 girls, 5 boys  ***Race/ethnicity:*** Not stated | ***Care type:*** Foster care  ***Time in care:*** 3-17 years  ***Time in current placement:*** Not stated  ***Number of placements:*** Not stated |
| Rogers 2018  England | Presents findings from research into how young people growing up in foster care in the UK manage the relationships in their social networks and gain access to social capital | Interviews and photo elicitation methods | 10 | ***Age:*** 12-14  ***Gender or sex:*** 5 female, 5 male  ***Race/ethnicity:*** 8 identified as White British, 2 identified as dual heritage, White  British and Caribbean. | ***Care type:*** Foster care  ***Time in care:*** At least 6 months  ***Time in current placement:*** Not stated  ***Number of placements:*** Unclear |
| Dansey 2018  England | Assesses the extent to which children in foster care experience loyalty conflict and how they cope with the challenges it presents | Semi-structured interview | 15 | ***Age:*** 7-14  ***Gender or sex:*** 8 females, 7 males  ***Race/ethnicity:*** 8 White British, 5 Black British, 2 Mixed Heritage | ***Care type:*** Foster care  ***Time in care:*** 1-13 years  ***Time in current placement:*** 1-9 years  ***Number of placements:*** 1-6 |
| Carr 2017  England* | Explores children’s relationships with animals in the context of foster care using attachment theory as a theoretical lens | Semi-structured interviews, guided diary | 8 | ***Age:*** 10-16  ***Gender or sex:*** 5 girls, 3 boys  ***Race/ethnicity:*** White | ***Care type:*** Foster care  ***Time in care:*** 4-8 years  ***Time in current placement:*** 7-13 months  ***Number of placements:*** 6-10 |
| Pert 2017  England | Explores service users’  perspectives of looked after children (LAC) reviews | Interviews | 25 | ***Age:*** 8-17  ***Gender or sex:*** 13 girls, 12 boys  ***Race/ethnicity:*** 5 from minority ethnic background | ***Care type:*** Foster care  ***Time in care:*** 8 months-9 years (M=3 years)  ***Time in current placement:*** Not stated  ***Number of placements:*** Not stated |
| Goodyer 2016  England | Explores how children made sense of their experiences of joining new foster families | Semi-structured interviews | 22 | ***Age:*** 9-17  ***Gender or sex:*** 15 females, 7 males  ***Race/ethnicity:*** 18 identified as White British, 3 as Black British, 1 as mixed-race British | ***Care type:*** Foster care  ***Time in care:*** Not stated  ***Time in current placement:*** Not stated  ***Number of placements:*** Unclear |
| Skoog 2015  Sweden | Investigates how children who have experienced instability in care describe their sense of belonging and relationships with adults who share responsibility for caring for them | Interviews | 12 | ***Age:*** 8-18  ***Gender or sex:*** 6 girls, 6 boys  ***Race/ethnicity:*** Not stated | ***Care type:*** 4 foster care; 3 foster care, institution, and secure units; 2 kinship care; 1 foster care and institution; 1 foster care and kinship care; 1 foster care, institution, and consultant care  ***Time in care:*** Unclear  ***Number of placements:*** 1-11 |
| Madigan 2013  Scotland | Contributes to our understanding of the impact of the context of foster care on young people’s representations of ‘feeling the same or feeling different’ and considers how this relates to the extant literature on identity development | Interviews | 9 | ***Age:*** 12-16  ***Gender or sex:*** 4 female, 5 male  ***Race/ethnicity:*** Not stated | ***Care type:*** Foster care  ***Time in care:*** Placement length 7 months-4 years  ***Number of placements:*** 4 participants had multiple placements |
| Ponciano 2013  United States | Explores the competence of youth in foster care to conduct PAR [participant action research] and discovers if PAR uncovers common experiences and strengths amongst youth in foster care | Interviews during Walk-the-Line Game | 16 | ***Age:*** 13-17  ***Gender or sex:*** 44% female, 56% male  ***Race/ethnicity:*** 81% identified as African American, 6% as Latino American, and 13% as White | ***Care type:*** Foster care  ***Time in care:*** Not stated  ***Time in current placement:*** Not stated  ***Number of placements:*** Not stated |
| Pölkki 2012  Finland | Examines the quality of foster children’s participation, as well as mechanisms that promote or hinder children’s participation in different phases and contexts of child-protection services | Semi-structured interviews | 8 | ***Age:*** 7-17  ***Gender or sex:*** Not stated  ***Race/ethnicity:*** Not stated | ***Care type:*** Foster care  ***Time in care:*** 1-14 years  ***Time in current placement:*** Unclear  ***Number of placements:*** 1-3 |
| Morrison 2011  Canada | Explores the perspectives of  children, foster parents and child protection workers regarding supervised access visits for Crown wards in Ontario, Canada | Semi-structured interviews | 24 | ***Age:*** 8-12 years (M=10.8 years)  ***Gender or sex:*** 11 girls, 13 boys  ***Race/ethnicity:*** Not stated | ***Care type:*** Foster care  ***Time in care:*** Not stated  ***Time in current placement:*** Not stated  ***Number of placements:*** Not stated |
| Rostill-Brookes 2011  England | Considers how young people, foster carers and social workers made sense of unplanned placements’ endings | Interviews | 5 | ***Age:*** 9-15  ***Gender or sex:*** 2 males, 3 females  ***Race/ethnicity:*** 3 White British, 2 dual heritage | ***Care type:*** Foster care, 1 currently in residential care  ***Time in care:*** 4-12 years  ***Time in current placement:*** Not stated.  ***Number of placements:*** 2-6 |
| Mitchell 2010a, Mitchell 2010b  Canada | Considers how children experience this potentially significant life transition [into foster care], to identify significant events in this transition from children's point of view, and to explore children's interpretations and evaluations of these events | Semi-structured interviews | 20 | ***Age:*** 8-15 (M=12)  ***Gender or sex:*** 13 females, 7 males  ***Race/ethnicity:*** Not stated | ***Care type:*** Foster care  ***Time in care:*** 6-36 months (M=20 months)  ***Time in current placement:*** Unclear  ***Number of placements:*** Not stated |
| Winter 2010, 2014  Northern Ireland | Presents the findings of research with young children in care (aged 4–7 years) regarding their views on their circumstances and care experiences | Semi-structured interviews | 14 | ***Age:*** 4-7  ***Gender or sex:*** 5 female, 9 male  ***Race/ethnicity:*** Not stated | ***Care type:*** 10 foster care, 2 currently at home  ***Time in care:*** 6 months-5 years and 6 months  ***Time in current placement:*** Not stated  ***Number of placements:*** 9 children had more than 3 house moves in 1 year |
| Daly 2009  Australia | Involves children and young people who have experienced living in foster care in the design of a research study focusing upon their views on what makes a good foster carer | Semi-structured interviews and focus groups | 14 | ***Age:*** 8-21 (6 8-12 yr olds, 7 13-17 yr olds, 1 21 yr old)  ***Gender or sex:*** 7 females, 7 males  ***Race/ethnicity:*** Participants were from a range of  cultural backgrounds including Aboriginal, Torres Strait Islander, and Australian South Sea Islander. | ***Care type:*** Foster care  ***Time in care:*** Not stated  ***Time in current placement:*** Not stated  ***Number of placements:*** Not stated |
| Bogolub 2008  United States | Gathers first-person data on the initial step in the removal–placement trajectory | Interviews | 6 | ***Age:*** 9-16  ***Gender or sex:*** 4 girls, 2 boys  ***Race/ethnicity:*** 2 identified as African American, 1 as Latina, 3 as White | ***Care type:*** Foster care  ***Time in care:*** 1-5 months  ***Time in current placement:*** 1-5 months  ***Number of placements:*** Not stated |
| Warming 2006  Denmark | Gives voice to foster children so that their knowledge about “the life as a child in foster care” could find its way to social workers and foster parents as well as politicians, thus influencing, democratizing and qualifying social work with children and youth in foster care | Individual open-ended interview, group discussions, establishment of a web-based “foster children’s public forum” | 15 | ***Age:*** 10-13  ***Gender or sex:*** Unclear  ***Race/ethnicity:*** Not stated | ***Care type:*** Foster care  ***Time in care:*** Not stated  ***Time in current placement:*** Not stated  ***Number of placements:*** Not stated |
| Mosek 2004  Israel | Explores the significance of the relationship between the foster care service model and experiences of the partners in care | Semi-structured interviews | 39 | ***Age:*** 5-18  ***Gender or sex:*** 21 boys, 18 girls  ***Race/ethnicity:*** Not stated | ***Care type:*** Foster care  ***Time in care:*** Not stated  ***Time in current placement:*** Not stated  ***Number of placements:*** Not stated |
| Singer 2004  Netherlands | Discusses a study of children’s perspectives on disciplinary conflicts with their foster parents | Semi-structured interviews | 45 | ***Age:*** 8-13  ***Gender or sex:*** 27 females, 18 males  ***Race/ethnicity:*** 26 Native Dutch, 18 Migrant, 1 unsure | ***Care type:*** Foster care  ***Time in care:*** Not stated  ***Time in current placement:*** 80% lived with recent foster family for 2 years or longer  ***Number of placements:*** 75% had 1 to 2 placements, 25% had 3 or more placements |
| Whiting 2003, Lee 2007  United States | Allows children who are currently in foster care tell their life stories as broad pictures, including early memories, current circumstances, and thoughts about the future | Semi-structured interviews | 23 | ***Age:*** 7-12 (M= 9.6 years)  ***Gender or sex:*** 7 girls, 16 boys  ***Race/ethnicity:*** 19 African American, 1 biracial, 3 White | ***Care type:*** Foster care  ***Time in care:*** M=36 months  ***Time in current placement:*** Not stated  ***Number of placements:*** 1-8 |
| Munro 2001  England | Ascertains from children their views on being looked after and the degree of power that they felt they had to influence decisions made about them | Interviews | 15 | ***Age:*** 10-17  ***Gender or sex:*** 7 girls, 8 boys  ***Race/ethnicity:*** 7 Black, 8 White | ***Care type:*** 13 foster care, 2 residential care  ***Time in care:*** 2-13 years  ***Time in current placement:*** Not stated  ***Number of placements:*** Not stated |
| *****Study participants listed as from “the UK,” primary study author resides in England. | | | | | |
