# Supplementary material for: Foster children’s perspectives on participation in child welfare processes: A meta-synthesis of qualitative studies
Source: PLoS One. 2022 Oct 10;17(10):e0275784. doi: 10.1371/journal.pone.0275784 (PMC9550086; doi:10.1371/journal.pone.0275784)
Supplement: S4 Table — (DOCX) [file pone.0275784.s004.docx]

**S4 – Facets of Participation**

| **Facets of participation** |  |  |  |
| --- | --- | --- | --- |
| **Subtheme** | **Theme** | **Subtheme** | **Countries represented by illustrative quotes**^a^ |
| **I wasn’t told**   - “nobody explained to me”(1); “no-one said”(2); “they never told us”(2); “they should tell me”(3); “nobody wants to tell you nottin”(4); “I don’t really get told much about my life”(5); “He promised me that he would talk to me in person and never did!”(6) | **Children are TOLD (by adults)** | **I was told**   - “have explained and told”(1); “(social worker) said”(1); “They told me”(3); “they said”(6); “talk to children”(7); “talks to me beforehand” (7); “told me”(8) | Australia (5), England (2,3,7,8), Canada (4), Finland (1), United States (6) |
| **I wasn’t prepared**   - “They just came to school and took me”(2); “I come home one night and seen my bags sitting in the hall and she just said ‘you’re moving’”(2); “we got lost on the way here [to carers]”(2); “how will I get back?”(2); “Well I start getting nervous kind of thing cos I’ve got to go to a new home, new school and stuff and I don’t know anyone”(3); “I just moved the same day”(3); “I got tooken”(4); “It’s like you’re being kidnapped”(4); “Woah!…Who are these people? I don't even know them and I'm moving in with them”(4); “I'll get locked up as long as I'm not with just some family I don't know”(4); “they said hurry, hurry up, get out of the tub. So we got our clothes on, and my mamma was crying and we was too, so they just rushed us out of the house…it was scary but I was just crying.”(6); “She perceived she was ‘dumped’ with people she had never met before in a place she had ‘never flipping heard of', far from her friends” (9) | **Children are PREPARED (by adults)** | **I was prepared**   - “I came here on a visit after the meeting. It was nice, Friday when I came, me and Jane (foster child) was watching TV and we went swimming and after that I went back to Tina (previous foster carer) and after that we came here”(2); “We came here and stayed for about two weeks, like a holiday, then we went back and got ready to live here” (2) | Canada (4), England (2,3), Scotland (9), United States (6) |
| **I didn’t/don’t know**   - “What do you mean?”(1); “I did not understand” (1); “I had not understood”(1); “I didn’t know”(10); “I don’t know why”(11); “I didn’t even know”(2); “I want to see my mom more often because I am a little worried about her”(12); “does anyone know what’s going on”(4); “I thought when I first went into foster care that I wouldn’t be able to see my parents again”(4); “Guys, help me make some sense out of this”(4); “I didn’t know where I was going to sleep”(4); “I don’t know”(13); “I want to know”(14); “I wish I knew”(6); “I don’t know”(6); “who are they?”(6) | **Children KNOW** | **I knew/know**   - “I understand”(1); “it meant that”(1); “My family was like a war”(10); “I knew” (2); “it was because”(12); “knowing what to expect”(3); “it is good to know that I won't have to move”(15) | Canada (4,11), England (2,3,10), Finland (1), Israel (14), Sweden (12,15), United States (6,13) |
| **I wasn’t asked**   - “I have never been asked”(1); [Interviewer]: “And you know when they were making those rules…did they ask what your opinion was, did they get your view?” [Child]: “No.” I: “What do you think about that?” [Child]: “Don’t like it. That’s why I went bonkers.” (16) | **Children are ASKED (by adults)** | **I was asked**   - “they have always asked”(1); “when they ask”(1); “asks how one feels”(17); “Ask them how are they feeling about this place they’re living at now and would they change anything”(5); “when people ask me”(8); “she asked me questions about how I was doing”(15) | Australia (5), Denmark (17), England (8), Finland (1), Northern Ireland (16), Sweden (15) |
| **I didn’t/couldn’t talk**   - Answering: “I cannot really always answer”(1); - Asking: “I myself could not ask anything”(1); “next time I won’t even ask”(18); “I don’t have the power to ask”(19) - Talking: “I can’t really say it”(16); “I don’t talk to anyone” (2); “But at first I didn't start talking to her”(4); “I try not to talk to them”(18); “I’d better keep it quiet”(18); “If you start whining, you only get in trouble”(18); “I don’t know how to explain”(8) | **Children TALK** | **I talk**   - Asking: “I always ask”(1); “ask your worker”(20) - Talking: “If you say something…”(17); “I can tell”(17); “Those times that I have talked”(12); “now we have heart-to-heart conversations”(4); “They (the children) might have good ideas”(5); “children that are in care are actually having a say about where they will live and that sort of thing, the people that they’ll live with and what good qualities that foster parents have”(5); “then when they like open their mouth and tell people”(5); “‘I can give more ideas”(5); “they should let us”(13); “I am going to talk”(14); “been talking to her” (7); “when talking to people”(8); “she helps me to say things”(21); “tell your counselor”(20); “to tell people”(9); “I talk”(15); “don’t say, ‘Oh, he is black [sic]; he is not allowed to enter my home’; don’t be racist”(22) | Australia (5), Canada (4,20), Denmark (17), England (2,7,8,21), Finland (1), Israel (14), Netherlands (18,22), Northern Ireland (16), Scotland (9), Sweden (12,15), United States (13,19) |
| **They don’t/didn’t listen**   - “When you try to correct some misunderstanding by trying to say what really happened, [they] say that it is my opinion…”(1); “they don’t wanna hear what they’re saying”(23); “No, I don’t think that she [the social worker] would listen”(17); “they just won’t listen”(2); “they can’t manage really listening”(12); “they don’t listen”(3) | **Adults LISTEN to children** | **They listened**   - “taken me into account”(1); “she listens”(1); “It’s about time somebody listens to us…”(17); “listen to your ideas”(20) | Canada (20), England (2,3), Denmark (17), Finland (1), Northern Ireland (23), Sweden (12) |
| **I didn’t/couldn’t participate/decide**   - Participating: “I have never participated in…”(1); “I haven’t really had involvement”(5); - Deciding: “It is really oppressive when they [adults] say…yes, you have to go outside”(1); “she just makes the decisions. There are rules that I have to obey”(16); “I found out I was going to have to”(12); “they didn’t give me enough freedom…I wasn’t allowed”(3); “I just get told what to do pretty much”(5); “I cannot change my mother’s [foster carer] mind…she won’t let me anyway.”(18); “If I was bad there they would give me a shot in the butt . . . and I got one, too”(6); “she won’t let me go”(8) | **Children PARTICIPATE or DECIDE** | **I participated/decided**   - Participating: “You’re able to help the others with their troubles, or they’re able to help you”(17); “I’ve taken part”(17); “I’ve been to a few of my placement meetings to find out what happened to me. I only go to those sometimes and that’s pretty much my involvement”(5) - Deciding: "Well, I got used to it, coz if I got used to it and I settled down quickly . . . if I didn’t, I would be in another place right now"(10); “It’s my decision”(11); “They was meant to be my carers, I was only there for a few days, they didn’t work out, and then I went, you know what I mean? I ran away to my mum’s, I always went to my mum’s, my sister’s, so . . . I hated the people I was with, awful, horrible, but I like it here.”(2); “I do talk to my aunties and uncles on the phone sometimes but I mainly like to keep the phone to my mum and my nan, and sometimes my friends but we will mainly BBM [Blackberry messaging] and stuff”(24); “I just thought, tell the social worker, so she come round later and I just got to move”(2); “Let them choose what they want for supper. Let them have video game things if they like video games.” (20); “I can decide”(15); “let them [foster youth] have foreign friends”(22) | Australia (5), Canada (11,20), Denmark (17), England (2,3,8,10,24), Finland (1), Netherlands (18,22), Northern Ireland (16), Sweden (12,15), United States (6) |
| ^a^Quotes in studies were inconsistently labelled with some factors (e.g., age, gender of child) and rarely labelled with other factors (e.g., ethnicity, placement factors). As such, we were not able to conduct analysis of themes using these elements. However, we have included the country and age sample of the study as a whole to give some context to the themes.  ^b^The age range of sampled children in studies included school-aged children (ages 6 to 12), adolescents (ages 13 to 17), or both (between ages 6 to 17). Only two studies investigated children under seven (14,23). | | | |

**Table explanation:**

In the table, partial or full quotes of children’s words are used to explain each sub-theme; for example, in the sub-theme “I was told” children might say adults “explained” or “told” them about aspects of foster care.

1. ***Foster children know only when they are told or prepared***

It was common for children to express that they *didn’t know* (at the time of entering care) or currently *don’t know* about aspects of foster care. Things children didn’t/don’t kno*w* about included their personal history prior to foster care (if placed at a young age); what placement or foster care means; what their foster family will be like; why they became involved with child protection services, including social workers; when they would get to see their biological family; how their family was doing (e.g., worrying about their biological family); where their siblings were placed; why their visits with their family were scheduled as they were (e.g., many had concerns about both frequency and duration of visits); when they would be able to return home; why they were moved from one placement to another; whom they could contact if things were not going well in their foster care home; and what would happen to them in the future. Some children didn’t know the name of their case workers. Not knowing was very stressful for many children, some expressing that they didn’t know where they would sleep, where they were geographically (if they could find their way back home), if the foster family would have toys, or if the foster carers would hit their siblings.

It was less common for children to describe *knowing* about aspects of foster care, such as the names of their carers before they were placed. Some children expressed that they *knew* why they were in foster care, but knowing could be characterized as self-blame:

Interviewer: Do you know why you don’t live with your mummy, daddy…? [Child]: Yep Interviewer: And why is that? [Child]: Cos I was cheeky to them. (23)

Other children described how they were placed because of difficulties at home (e.g., “my family was like a war” (10)) or because they weren’t happy (“I didn’t really like it there”) (23). When children did know about aspects of foster care, it was usually because an adult in their life had told them, such as a social worker, their parent or relative, or their foster carer. One child expressed that they knew what placement was because they asked a friend (“I did not understand what a placement meant. Then I asked my friend” (1)).

In most cases, children spoke positively about being *told* (“They [social workers] have always explained and told and explained in a way that I have understood” (1)) and negatively about not being told (“nobody wants to tell you nottin” (4)). However, some children were told about aspects of foster care by strangers; for example, one child said, “The taxi told me”(4)). Another child was told that they were moving to a new placement by a stranger:

Interviewer: All right, so when did you know that you were going to have to go? [Child]: On the same day, on the night. Interviewer: On the night. Ok and who told you about that? [Child]: Umm, this man that came. I don’t know this man. He was umm, begins with e-d-t or something like that. (3)

One child also discussed how they didn’t want to be told *again* because they already knew: “if they already know about everything why they’re here, why they’re going there, then don’t bring it up again because if I already knew and they brought it up again, it kept running through my mind” (20).

Aside from the stress about not knowing, or not being told about important aspects of their lives, children particularly found not being *prepared* for removal from home to be a stressful or traumatic experience. For example, one child described this experience as being “kidnapped” (4) and another child described being rushed out of the bath while their mother cried (6). Stressful appraisals were also connected to having to live with strangers (i.e., foster carers): “I'll get locked up as long as I'm not with just some family I don't know” (4). In contrast, only two children in one study described examples of being prepared for a move between placements. These examples had a more leisurely tone, as one child described a visit that involved swimming (2) and another child described the visit as a holiday (2).

1. ***Conversations with foster children: Adults ask, children talk, adults listen***

Children described ideal and less ideal conversations with adults about aspects of foster care. Most children who had been *asked* about aspects of foster care described this positively (“They have always asked how things are and encouraged me to speak in confidence…” (1)). One child, however, discussed how they didn’t know what to say when asked a question by an adult: “I find the [care review] meetings rather unpleasant, when they ask what I think of this and that matter. I cannot really always answer that kind of question.” (1)

In contrast, two children expressed anger or disappointment that they were not asked about their opinion or about aspects of their lives in foster care, including one child who indicated that this made them ‘bonkers’:

[Interviewer]: “And you know when they were making those rules…did they ask what your opinion was, did they get your view?” [Child]: “No.” I: “What do you think about that?” [Child]: “Don’t like it. That’s why I went bonkers.” (16)

Children described *talking* or not talking to adults by answering questions, asking questions, or sharing their views (explaining, talking, saying, speaking). There were many examples where children expressed the belief that they could or could not share their views. Children also described examples when adults did or did not *listen* to them. These examples of talking/not talking and adults listening/not listening were closely tied to barriers and facilitators of participation, which are discussed in supplemental file 5.

1. ***Foster children participate and decide***

Finally, compared to examples of conversations with children (adults asking, children speaking, adults listening), there were less examples where children described situations where they could or could not *participate* in decisions or activities. Situations where children could not participate were often linked to rules in the foster care home that they disagreed with. In contrast, situations where children could participate were often linked to participatory activities set up by adults. For example, in the study conducted by Warming (17) children discussed how they enjoyed participating in writing a book or writing about “whatever they wanted” on a web-based forum set up for foster children. Children also discussed participating or not participating in their care reviews.

In terms of *decisions*, two children described deciding to connect with biological family members in some way (e.g., over Facebook) (24), another child described deciding to talk to their social worker about their placement (2), and one child from one study (16) and five children from another study (2) discussed deciding to run away from their foster home. In contrast, eight children across studies (3–6,12,16,18) expressed frustration with not being able to decide about aspects of their foster care experience, such as being told to wait outside during their care review, not getting to decide if and when they moved placements, or not getting a say in any rules or decisions in their foster care home.

**References**

1. Pölkki P, Vornanen R, Pursiainen M, Riikonen M. Children’s participation in child-protection processes as experienced by foster children and social workers. Child Care in Practice. 2012 Apr 1;18(2):107–25.

2. Goodyer A. Children’s accounts of moving to a foster home. Child & Family Social Work. 2016;21(2):188–97.

3. Rostill-Brookes H, Larkin M, Toms A, Churchman C. A shared experience of fragmentation: Making sense of foster placement breakdown. Clinical Child Psychology and Psychiatry [Internet]. 2010 Jun 10 [cited 2020 Jul 8]; Available from: https://journals.sagepub.com/doi/10.1177/1359104509352894

4. Mitchell MB, Kuczynski L. Does anyone know what is going on? Examining children’s lived experience of the transition into foster care. Children and Youth Services Review. 2010 Mar 1;32(3):437–44.

5. Daly W. “Adding their flavour to the mix”: Involving children and young people in care in research design. Australian Social Work. 2009 Dec 1;62(4):460–75.

6. Whiting JB, Lee RE. Voices from the system: A qualitative study of foster children’s stories. Family Relations. 2003;52(3):288–95.

7. Munro E. Empowering looked after children. Child and Family Social Work. 2001 May;6(2):129–37.

8. Carr S, Rockett B. Fostering secure attachment: experiences of animal companions in the foster home. Attach Hum Dev. 2017 Jun;19(3):259–77.

9. Madigan S, Quayle E, Cossar J, Paton K. Feeling the same or feeling different? An analysis of the experiences of young people in foster care. Adoption & Fostering. 2013 Dec 1;37(4):389–403.

10. Dansey D, John M, Shbero D. How children in foster care engage with loyalty conflict: presenting a model of processes informing loyalty. Adoption & Fostering. 2018 Dec 1;42(4):354–68.

11. Morrison J, Mishna F, Cook C, Aitken G. Access visits: Perceptions of child protection workers, foster parents and children who are Crown wards. Children and Youth Services Review. 2011 Sep 1;33(9):1476–82.

12. Skoog V, Khoo E, Nygren L. Disconnection and dislocation: Relationships and belonging in unstable foster and institutional care. Br J Soc Work. 2015 Sep 1;45(6):1888–904.

13. Bogolub E. Child protective services investigations and the transition to foster care: Children’s views. Families in Society: The Journal of Contemporary Social Services. 2008 Jan 1;89(1):90–9.

14. Mosek A. Relations in foster care. Journal of Social Work. 2004 Dec 1;4(3):323–43.

15. Wissö T, Johansson H, Höjer I. What is a family? Constructions of family and parenting after a custody transfer from birth parents to foster parents. Child & Family Social Work. 2019;24(1):9–16.

16. Winter K. Understanding and supporting young children’s transitions into state care: Schlossberg’s transition framework and child-centred practice. Br J Soc Work. 2014 Mar 1;44(2):401–17.

17. Warming H. “How can you know? You’re not a foster child”: Dilemmas and possibilities of giving voice to children in foster care. Children, Youth and Environments. 2006;16(2):28–50.

18. Singer E, Doornenbal J, Okma K. Why do children resist or obey their foster parents? The inner logic of children’s behavior during discipline. Child Welfare. 2004 Dec;83(6):581–610.

19. Ponciano L. The voices of youth in foster care: A participant action research study. Action Research. 2013 Dec 1;11(4):322–36.

20. Mitchell MB, Kuczynski L, Tubbs CY, Ross C. We care about care: advice by children in care for children in care, foster parents and child welfare workers about the transition into foster care. Child & Family Social Work. 2010;15(2):176–85.

21. Pert H, Diaz C, Thomas N. Children’s participation in LAC reviews: a study in one English local authority. Child & Family Social Work. 2017;22(S2):1–10.

22. Degener CJ, van Bergen DD, Grietens HWE. The ethnic identity complexity of transculturally placed foster youth in the Netherlands. Children and Youth Services Review. 2020 Jun 1;113:104957.

23. Winter K. The perspectives of young children in care about their circumstances and implications for social work practice. Child & Family Social Work. 2010 May 1;15(2):186–95.

24. Rogers J. Preserving and memorialising relationships: exploring young people’s experiences of foster care through the lens of social capital. Adoption & Fostering. 2018 Jul 1;42(2):176–88.
