# Supplementary material for: Foster children’s perspectives on participation in child welfare processes: A meta-synthesis of qualitative studies
Source: PLoS One. 2022 Oct 10;17(10):e0275784. doi: 10.1371/journal.pone.0275784 (PMC9550086; doi:10.1371/journal.pone.0275784)
Supplement: S5 Table — (DOCX) [file pone.0275784.s005.docx]

**S5 – Facilitators and barriers of foster children’s participation**

| **Barrier to participation** | **Theme** | **Facilitator of participation** | **Countries represented by illustrative quotes**^a^ |
| --- | --- | --- | --- |
| **Individual qualities** |  |  |  |
| Ten children within and across several studies described not being able to participate due to aspects of themselves (their thoughts, emotions, and behaviours), such as a general sense of not being able to answer questions about themselves (1,2); a sense that they could not answer about aspects of foster care because they were actively trying not to think about being in care (3), because they were so angry (4), “repressing” their feelings (4), or trying not to get into a fight (4).  Three children described examples of their wishes not coming true, such as wishing for a better ending at a placement (5), wishing that fostering did not happen (or that they could live with both families) (6), or wishing they knew where their siblings where:   - “I don’t get to see my brothers and we’re all split up…yeah, I…wish I knew where they were” (7). | **Thoughts, feelings, behaviours, and wishes**  Children described how their own thoughts, emotions, and behaviours affected their participation in aspects of foster care. Children also described how their wishes related to foster care did not come true. | Two children described knowing “what’s best” for them in terms of cancelling visits with the biological family (e.g., if sick) (8) or recognizing that they were more effective in their communication with adults when they were calm (9). | Canada (8,9), England (2,3), Finland (1), Israel (6), Netherlands (4), Sweden (5), United States (7) |
| Five children across studies (1,4,10–12) described a sense of powerlessness—not being able to ask or not knowing what to do:   - “I myself could not ask anything. I just went with them [social workers].” (1) - "I don't do anything at all. I don't know what to do.” (4) | **Self-efficacy**  Children discussed their belief in their ability to effect change in their life or produce desired results. | Two children (3,8) described having a strong sense that it was important to make decisions:   - “Don’t be stuck in the middle. . .think what you would like to do; it is not their decision in life, it is your decision that you have to make” (3). | Canada (8), England (3,10,11), Finland (1), Netherlands (4), Northern Ireland (12) |
| **Who told foster children (informants), what children were told (developmental appropriateness, options), when they were told (timing), and how (access to technological means)** | | |  |
| In several studies (5,8,13–15) children discussed being told about aspects of foster care by strangers, such as taxi drivers (15) or workers they had just met (5,13,14). Children also described situations where they hoped to change things about foster care but did not know whom to contact (8). | **Informants**  Children discussed the people who communicated information about foster care to them. | Two children in one study discussed how the informant was someone they had a relationship with, such as their parents, their social worker, or their foster carer:   - “People like social workers and foster mother and mother have told me. I do not remember exactly who has told me the most” (1). | Canada (8,15), England (13,14), Finland (1), Sweden (5) |
| One study described children reading documents related to foster care that they found confusing or frightening (1). | **Developmental appropriateness**  Children discussed if they could understand the information that was given to them about foster care. | Two children (1,11) described the issue of whether they were able to understand information given to them by informants. One child expressed adults communicating in a way they understand:   - “They [social workers] have always explained and told and explained in a way that I have understood” (1). | England (11), Finland (1) |
| Children in three studies (5,11,16) described situations where they felt they had no choices. One child discussed not having a choice about where they were placed (on a farm, in a facility that included a twelve step program (5)) and another child discussed not having a choice about when their care review happened because it revolved around adults’ schedules:   - “It’s [care review] always after school when I’m tired and everyone else is going to the shops and hanging out and that and I’m like oh, I’ve got to go home…but they stop work at 5 so it has to be then, doesn’t it?” (11).   Another child discussed not feeling they had choices and how this meant they had to live on the streets:   - Interviewer: “Where do you think you should be now?” [Child]: “I think I should be on the streets.” Interviewer: “Do you? . . . You think you should be on the streets?” [Child]: “Well, I can’t go home and I can’t go and I don’t want to go to another foster placement.” (12) | **Choices**  Children discussed if they had a choice in matters of importance to them. | One study reported that a child had been given the option of counseling and that this had improved their relationships with others (5). In another study a child requested that children be given choices for what school they attend:   - “Give the youth an opportunity to visit a whole bunch of different schools and then choose which one they want to go to” (9). | Canada (9), England (11), Northern Ireland (12,16), Sweden (5) |
| One child described how they should be told about aspects of foster care *before* they happen:   - “No-one said anything before…” (13).   Another child felt that they would be able to resolve things in their foster home if they had been given advanced warning:   - “Well [pause] they should tell me simply or plain that I’m moving cos then I could go, ‘Ok, what have I done wrong’ and probably correct it before they put anything in and I could probably go fine” (14). | **Timing – before vs after**  Children commented on the timing of when something in foster care should occur. |  | England (13,14) |
| Eleven children from several studies (3 children each from two studies (13,14), two children from one study (15) and one child each from three studies (5,7,17)) described decisions that didn’t happen in advance, which gave children the sense that they were rushed (including rushed in saying good-bye to family members). In some cases, children described the stressful decisions being communicated to them last minute:   - “I didn’t know until I was half way there, cos then she [social worker] told me. She had a phone call in the car saying they’ve got me a place and that was it. So, umm cos that was nerve wracking cos it was seven o’clock at night and I had nowhere to go.”(14)   For some children, however, study authors described how children seemed numb to last-minute changes that they (the study authors) felt were “shocking”:   - Interviewer: So when you say your foster carers didn’t tell you? [Child]: I just moved one day. I woke up and all my bags were packed. (14) | **Timing – rushed vs leisurely**  Children discussed if adults gave them sufficient time to prepare them for changes in their lives. | Two children in one study (13) reported more positive experiences of transitions between placements when they were prepared:   - “I just remember coming for one day to see if I liked them, but no more, that’s all. I felt right at home, right away…” (13). - We ([child] and his social worker) came down on the train and we had lunch here” (13). | Canada (15), England (13,14), Scotland (17), Sweden (5), United States (7) |
|  | **Access to technology** | Five children (three from one study (18)) described how having access to computers, social media (Facebook), and smartphones (e.g., BBM) was important to connecting with important people in their lives:   - “I don’t see her but Facebook helps me stay in touch with her.” (19) | England (19), Sweden (18) |
| **Qualities of relationships that foster children found important when participating** | | |  |
| Two children from one study (4) and six children across studies discussed interactions where they felt their sharing was not appreciated because they might say bad words (16), or they believed their carers or social workers thought they were a pest (12), spoiled (20), or a baby (4). Some children also felt they should not share because their sharing got them in trouble (13) or they might be perceived as whining (4). One child conveyed that adults only talked to them when they were in trouble and did not notice the “things you do right” (5). | **Appreciated**  Children discussed their sense of being respected, valued or appreciated when they participated. | One child discussed how foster children should be respected: “[T]reat the child with respect and good care” (9). Another child talked about the importance of her birth mother showing interest in her activities while she was in foster care: “It feels like she is interested in what I do” (18). | Canada (9), Denmark (20), England (13), Netherlands (4), Northern Ireland (12,16), Sweden (5) |
| Five children in three studies (5,10,13) described social workers rushing off to other meetings (13), barely calling them (5), and going on long vacations, thereby putting the child’s life on hold (5). One child also discussed feeling neglected by social workers (10). | **Availability**  Children discussed their perception of whether important people such as foster carers and particularly their social worker was available to them. | One child talked about how it was important for a social worker to “phone regularly” so they could “keep in touch with what is happening” (10). | England (10,13), Sweden (5) |
| Eight other children across studies (4,7,13,17,20,21) discussed difficulties with feeling they did not belong. One child talked about how it took a while for them to have a sense of belonging in foster care (20); another child discussed how they were afraid to share about themselves because they were afraid of being laughed at and disowned (4). One child closely connected a sense of not belonging as a barrier to participation:   - “I know that I had emphasized rights myself, but rights are not everything. You have to be careful. What does it help to know about your rights if nobody cares about you and you feel lonely, sad and excluded from everywhere.” (20)   One child talked about not belonging due to differences in class (“they’re posh”) (17) and a few children discussed how different race/ethnicity placements made them feel they did not belong:   - “My first placement, I didn’t get on [. . .] I didn’t want to come in. The other foster kid was black, I was the only white person there and I didn’t feel that comfortable. I just got up, got showered and went out.” (13) - “I’d rather be with a Black foster parent, no offense, but because people say, ‘ha ha, you got a White mom.’”(7) - One child felt like she had trouble belonging in either foster care or with her birth family because her foster carer commented on her ability to speak Moroccan (“Why do you talk Moroccan…Why do you talk so fast” (21)) while her birth mother expressed disappointment in her for not speaking Moroccan the same as her birth family (“My (birth) mother says…she says it because she’s ashamed …(speaks softly)…. it’s a shame that you can’t even talk our way”) (21). | **Belonging**  Some children across studies discussed their personal sense of belonging in close relationships with important people in their lives (e.g., foster carers, social workers) or not belonging and how this affected their participation. | One child discussed how they can tell their carers “everything” and they’ll care because “I’m like her own child” (20). Another child similarly discussed how they could tell anybody that they were a foster child because they feel “more secure” because “if something did go wrong then I’ve got somewhere [sic] that I could tell” (17).  Another child expressed how it is important for carers to “demonstrate that they are happy to have you as their foster child” by asking how the child feels and making them feel welcome (20). Another child discussed how being in a group of foster children enabled children to support and help one another, making them feel they’re “not the only foster child” (20). One child felt it was important for foster carers to not “be racist” and “accept their foster child is a foreigner” and “let them…have foreign friends” as they felt their foster parents did (21). | Denmark (20), England (13), Netherlands (4,21), Scotland (17), United States (7) |
| Four children (two children each in two studies (5,14)) described situations where they did not have strong connections with foster carers, such as when a carer told a child “sorry but you’ve got to leave” (14). One child suspected that social workers were not actually allowed to connect with children:   - “I started to suspect that it’s not acceptable for there to be connections between social workers and their patients or clients” (5). | **Connection**  Children described if they felt connected to meaningful adults in their lives and how this affected their participation. | Five children (2,4,9,10,15) described many situations where they had strong connections with their foster carers and how this evolved over time:   - “Before I felt that I couldn't talk to like [my foster mother], but now me and [my foster mother] have heart-to-heart conversations like all the time” (15).   One child felt this connection was facilitated by foster carers asking about things that were important to children, such as their favourite colour or other aspects of their lives (9). Some children described how pets made them feel safe enough to communicate in foster care:   - “I do feel more confident when he [the dog] is around, like when people ask me what I think about stuff and when talking to people I feel better if he [the dog] is just ‘around,’ but I don’t need him all the time – just when it’s difficult” (2). | Canada (9,15), England (2,10,14), Netherlands (4), Sweden (5) |
| Two children (10,20) discussed what they felt were breaches of confidentiality. One child discussed how they did not talk to their social worker because they were worried what they said would be passed to their foster carer (20). Another child discussed how she would not have told anything to her worker if she knew everyone would find out:   - “I felt really let down because I thought I had been talking to her privately but I saw she had written it all down in the file for anyone to read. I wouldn’t have said anything to her if I had known she was going to do that” (10). | **Confidentiality**  Children discussed their sense of whether information they shared would be kept confidential or private. | Two children (1,10) discussed how their social worker or counselor told them about the limits of confidentiality:   - “She has told me what kinds of things she would pass on to my social worker so I know the rest of it will be kept secret” (10). | Denmark (20), England (10), Finland (1) |
| Two children from one study (1) discussed situations where they were intentionally excluded from aspects of foster care. One child discussed how they have “never been asked to participate” (1) and another conveyed that what adults say “behind my back” frightens them (1). | **Inclusivity**  Children described situations where they were meaningfully included or excluded in aspects of foster care. | Two children (1,10) discussed situations where they were intentionally included from aspects of their foster care experience. For example, one child discussed how their social worker “always asked whether I have something to ask and how I’m doing” (1). | England (10), Finland (1) |
| Six children in five studies (5,7,10,13,15) described situations where their participation was blocked due to a person’s lack of integrity or honesty. For example, one child discussed how they were told they were going to be in a particular foster home for only a few weeks but that was “years ago” (13) and another child discussed how they did not believe what their worker told them about why they had to move placements (13). Two children (5,10) complained that adults, including social workers and judges, do not do what they promise:   - “What I want to know is why doesn’t the judge keep his promises. He promised me that he would talk to me in person and never did!” (7). | **Honesty**  Children described situations where their participation was blocked due to the person’s lack of honesty. |  | Canada (15), England (10,13), Sweden (5), United States (7) |
| Four children (2,13,14,16) discussed aspects of foster care being unfair. One child said it was unfair that they were taken from their home (16), one said it was unfair to have to move and change schools (13), one child said it was unfair that no one listened to them about an unwanted placement change and move (14) and two children suggested it was unfair that they could not see their mom (2,16). | **Fairness**  Children discussed aspects of foster care that they found unfair, situations that involved lack of participation in their lives. |  | England (2,13,14), Northern Ireland (16) |
| Five children (1,16,18,20,22) described situations where adults were “oppressive” (1), they had no power to affect decisions in their own lives (4) or felt adults intentionally tried to convince them that they know better than the child:   - “Next they try to convince you that they know best. Finally they just decide what they had decided before talking to you. Hmm. Well, some are on the child’s side and do listen, but they are always the ones with the least power to make decisions.” (20)   Five (1,5,7,14,20) children described situations where they felt adults in their lives, especially social workers, were ineffective; for example, not realizing the extent of problems in their home before placement (1) (including workers asking about children’s lives in front of parents and children not feeling safe to respond (1)), and not being listened to about the need for placement (20). One child discussed how they felt like they were just “one in a hundred” to social workers:   - You’re like just one in a hundred for them. They can’t manage really listening much and it really seems like they are stressed the whole time and really don’t have time (5). | **Power**  Children described situations that reflected different aspects of power in their relationships: adults’ power over children (adults’ force, coercion, domination, control), power with (shared power that grows out of collaboration and relationships), and power to (adults’ power to effect change in children’s lives). | In one study that evaluated different opportunities for foster children to participate (20), a child discussed their sense that different opportunities to participate, such as youth-run forums, enabled them to feel that they “run the show” (20). Five children (5,10,13,17,20) also described situations where adults, including social workers, had the power to affect their lives in a positive way:   - “She would sort out anything that was bothering me” (10). | Denmark (20), England (10,13,14), Finland (1), Netherlands (4), Northern Ireland (16), Scotland (17), Sweden (5,18), United States (7,22) |
| Seven children in five studies (5,9,13,16,20), described situations where they felt misunderstood. One child discussed how adults only understand things about foster children if they have read about it:   - “If you say something that they don’t expect or they don’t have in their books about foster children, they get confused” (20).   One child discussed how people don’t understand how sad children feel:   - “Some people don’t understand how sad the children feel and they just won’t listen, and they just feel like everything’s alright and the child’s saying that everything’s alright, but they could be really upset and not just showing it like” (13).   One child felt that they were not known by the social worker (5) and another noted that adults did not “give a shit about my suggestions” (5). One child confessed that it was unlikely that adults could understand them:   - “Are you sure you’ll be able to understand what the children write…you’re not a foster child—how can you?” (20). | **Understanding**  Children discussed if they felt meaningful adults in their lives understood them when they participated. | Four children in three studies (1,9,23) talked about the importance of others understanding them and their thoughts, wishes, and needs. This included carers listening to them, which could help them feel better (23). Children also appreciated carers who listened to children’s likes and dislikes (9). | Australia (23), Canada (9), Denmark (20), England (13), Finland (1), Northern Ireland (16), Sweden (5) |
| Two children discussed a lack of trust with important adults in their lives or how trust is not immediate. For example, one child noted:   - “Just ‘cos they’re under social services, it doesn’t mean that you can trust them” (13)   Another child noted that they couldn’t talk to their carer “right when I went there [to foster care] because I couldn’t really trust her yet” (15). | **Trust**  Children discussed how trusting adults was difficult and how this affected their participation. |  | Canada (15), England (13) |
| ^a^Individual quotes were inconsistently labeled with age and gender of contributing children and rarely labeled with other factors (e.g., race, ethnicity, placement factors), so analysis by these factors was not possible. | | | |

**Table explanation:**

It is important to note the following points about the framing of these facilitators and barriers of participation. First, many children discussed several themes in one quote. For example, one quote addressed understanding (“understands her feelings”), availability (“she can always be contacted”), and helpfulness (“she is involved in the thing and really wants to help”) of a social worker (1). Second, many of these themes could be said to be about *power.* For example, the quote “I myself could not ask anything. I just went with them [social workers],” which is found under self-efficacy, is unmistakably about the lack of power the child has to affect decision-making about removal from the home. However, to enable some granularity in the themes and to account for children’s framing of themes, themes related to power were only labelled as such if children were more clearly identifying differences in power as their key concern. Third, and relatedly, we did not code themes as we might clinically, but according to the theme that best captured children’s expressed views. Fourth, in some cases, children described ways that their participation was blocked but not how their participation was enhanced in relation to a particular theme. For example, several children described the sense that their wishes never came true, situations where adults did not keep their promises, and situations that they described as unfair.

References

1. Pölkki P, Vornanen R, Pursiainen M, Riikonen M. Children’s participation in child-protection processes as experienced by foster children and social workers. Child Care in Practice. 2012 Apr 1;18(2):107–25.

2. Carr S, Rockett B. Fostering secure attachment: experiences of animal companions in the foster home. Attach Hum Dev. 2017 Jun;19(3):259–77.

3. Dansey D, John M, Shbero D. How children in foster care engage with loyalty conflict: presenting a model of processes informing loyalty. Adoption & Fostering. 2018 Dec 1;42(4):354–68.

4. Singer E, Doornenbal J, Okma K. Why do children resist or obey their foster parents? The inner logic of children’s behavior during discipline. Child Welfare. 2004 Dec;83(6):581–610.

5. Skoog V, Khoo E, Nygren L. Disconnection and dislocation: Relationships and belonging in unstable foster and institutional care. Br J Soc Work. 2015 Sep 1;45(6):1888–904.

6. Mosek A. Relations in foster care. Journal of Social Work. 2004 Dec 1;4(3):323–43.

7. Whiting JB, Lee RE. Voices from the system: A qualitative study of foster children’s stories. Family Relations. 2003;52(3):288–95.

8. Morrison J, Mishna F, Cook C, Aitken G. Access visits: Perceptions of child protection workers, foster parents and children who are Crown wards. Children and Youth Services Review. 2011 Sep 1;33(9):1476–82.

9. Mitchell MB, Kuczynski L, Tubbs CY, Ross C. We care about care: advice by children in care for children in care, foster parents and child welfare workers about the transition into foster care. Child & Family Social Work. 2010;15(2):176–85.

10. Munro E. Empowering looked after children. Child and Family Social Work. 2001 May;6(2):129–37.

11. Pert H, Diaz C, Thomas N. Children’s participation in LAC reviews: a study in one English local authority. Child & Family Social Work. 2017;22(S2):1–10.

12. Winter K. Understanding and supporting young children’s transitions into state care: Schlossberg’s transition framework and child-centred practice. Br J Soc Work. 2014 Mar 1;44(2):401–17.

13. Goodyer A. Children’s accounts of moving to a foster home. Child & Family Social Work. 2016;21(2):188–97.

14. Rostill-Brookes H, Larkin M, Toms A, Churchman C. A shared experience of fragmentation: Making sense of foster placement breakdown. Clinical Child Psychology and Psychiatry [Internet]. 2010 Jun 10 [cited 2020 Jul 8]; Available from: https://journals.sagepub.com/doi/10.1177/1359104509352894

15. Mitchell MB, Kuczynski L. Does anyone know what is going on? Examining children’s lived experience of the transition into foster care. Children and Youth Services Review. 2010 Mar 1;32(3):437–44.

16. Winter K. The perspectives of young children in care about their circumstances and implications for social work practice. Child & Family Social Work. 2010 May 1;15(2):186–95.

17. Madigan S, Quayle E, Cossar J, Paton K. Feeling the same or feeling different? An analysis of the experiences of young people in foster care. Adoption & Fostering. 2013 Dec 1;37(4):389–403.

18. Wissö T, Johansson H, Höjer I. What is a family? Constructions of family and parenting after a custody transfer from birth parents to foster parents. Child & Family Social Work. 2019;24(1):9–16.

19. Rogers J. Preserving and memorialising relationships: exploring young people’s experiences of foster care through the lens of social capital. Adoption & Fostering. 2018 Jul 1;42(2):176–88.

20. Warming H. “How can you know? You’re not a foster child”: Dilemmas and possibilities of giving voice to children in foster care. Children, Youth and Environments. 2006;16(2):28–50.

21. Degener CJ, van Bergen DD, Grietens HWE. The ethnic identity complexity of transculturally placed foster youth in the Netherlands. Children and Youth Services Review. 2020 Jun 1;113:104957.

22. Ponciano L. The voices of youth in foster care: A participant action research study. Action Research. 2013 Dec 1;11(4):322–36.

23. Daly W. “Adding their flavour to the mix”: Involving children and young people in care in research design. Australian Social Work. 2009 Dec 1;62(4):460–75.
