# Supplementary material for: Foster children’s perspectives on participation in child welfare processes: A meta-synthesis of qualitative studies
Source: PLoS One. 2022 Oct 10;17(10):e0275784. doi: 10.1371/journal.pone.0275784 (PMC9550086; doi:10.1371/journal.pone.0275784)
Supplement: S1 File — (DOCX) [file pone.0275784.s006.docx]

**S2 File** – **Example Search**

Database: Ovid MEDLINE(R) ALL <1946 to November 07, 2019>
Search Strategy:
--------------------------------------------------------------------------------
1     Foster Home Care/ (3552)
2     ("foster care" or "foster home?" or "kin care" or "kinship care").tw. (2486)
3     (out-of-home adj5 (placement? or care)).tw. (800)
4     (("looked after" or "in care") adj5 (child* or infant* or baby or babies or toddler* or preschool* or pre-school*or teen* or adolescen* or youth* or young people)).tw. (967)
5     (local authorit* adj5 care).tw. (182)
6     ((child protection or child protective) adj3 investigation?).tw. (73)
7     or/1-6 (6073)
8     qualitative research/ or hermeneutics/ or interview/ (78658)
9     (qualitative or interview* or focus group* or ethnograph* or phenomenolog* or hermeneut*).tw. (517304)
10     ((child* or toddler* or preschool* or pre-school* or teen* or adolescen* or youth* or young people*) adj5 (view?
or perspective? or voice? or perception? or experience?)).tw. (59503)
11     or/8-10 (579676)
12     7 and 11 (1234)
13     limit 12 to yr="2000 -Current" (1054)

Database: Ovid MEDLINE(R) ALL <1946 to February 02, 2021>
Search Strategy:
--------------------------------------------------------------------------------
1     Foster Home Care/ (3650)
2     ("foster care" or "foster home?" or "kin care" or "kinship care").tw. (2675)
3     (out-of-home adj5 (placement? or care)).tw. (891)
4     (("looked after" or "in care") adj5 (child* or infant* or baby or babies or toddler* or preschool* or
pre-school*or teen* or adolescen* or youth* or young people)).tw. (1084)
5     (local authorit* adj5 care).tw. (204)
6     ((child protection or child protective) adj3 investigation?).tw. (86)
7     or/1-6 (6502)
8     qualitative research/ or hermeneutics/ or interview/ (89475)
9     (qualitative or interview* or focus group* or ethnograph* or phenomenolog* or hermeneut*).tw. (573930)
10     ((child* or toddler* or preschool* or pre-school* or teen* or adolescen* or youth* or young people*) adj5 (view?
or perspective? or voice? or perception? or experience?)).tw. (66553)
11     or/8-10 (642687)
12     7 and 11 (1376)
13     limit 12 to yr="2019 -Current" (226)
