# Supplementary material for: Foster children’s perspectives on participation in child welfare processes: A meta-synthesis of qualitative studies
Source: PLoS One. 2022 Oct 10;17(10):e0275784. doi: 10.1371/journal.pone.0275784 (PMC9550086; doi:10.1371/journal.pone.0275784)
Supplement: Attachment — Submitted filename: PLOS review_July.27.2021.docx [file pone.0275784.s007.docx]

This is a very interesting and well-written manuscript on the qualitative experiences of children within foster-care systems. Congratulations to the authors for their work. I have included several recommendations that can help the authors to strengthen the manuscript prior to publication.

1. Line 37 page 3. Reference 11 is noted twice in the same reference string.
2. Row 43, page 3. It would be helpful to define what “child welfare processes” consist of in this context, particularly given this reviews covers a wide variety of contexts. Do these vary across country settings and how did the authors define it?
3. The final paragraph in the introduction can be strengthened by adding more detail about other systematic reviews that have been conducted within this area, and how this systematic review differs/adds to that knowledge base.
4. Also in the final paragraph of the introduction, it seems clear that the aim is to summarize children’s perspectives. However, the reader is left to wonder, to what purpose? Who can use the information from this review and how can they use it? How is this information applicable within a practice or policy setting?
5. Search strategy, line 74. Is there a reason why key informant interviews were not included in the strategy?
6. Within the results section, there should be more information (beyond a supplementary table) regarding the CASP findings/methodological rigor of the included studies beyond inclusion of the table. What elements were weak that might have implications for the results?
7. Within the results section, findings are lumped mainly into a single table, and the discussion begins with new information that actually appears that it should belong in the results. It would be helpful to include more detailed information beyond the table within the results section, and also a more detailed analysis of findings across different contexts. For example, the participants are included from a variety of developed country settings around the world wherein the foster care system likely vastly differs. It would be helpful to provide more detailed information by either continent (or preferably) country-setting to identify if there are specific challenges/opportunities within individual contexts.
8. In addition to the above, a more detailed understanding of how children experience the foster care system by race/ethnicity/minority status/age/gender would be very interesting.
