# Supplementary material for: Foster children’s perspectives on participation in child welfare processes: A meta-synthesis of qualitative studies
Source: PLoS One. 2022 Oct 10;17(10):e0275784. doi: 10.1371/journal.pone.0275784 (PMC9550086; doi:10.1371/journal.pone.0275784)
Supplement: Attachment — Submitted filename: Responses to Editor and Reviewers.docx [file pone.0275784.s008.docx]

We would like to thank the Editors and Reviewers for their thoughtful and constructive feedback, which we believe has improved the manuscript. Editor and Reviewer responses are found in bold below, with our responses underneath.

**Responses to editor:**

**1. Please ensure that your manuscript meets PLOS ONE's style requirements, including those for file naming. The PLOS ONE style templates can be found at**

**https://journals.plos.org/plosone/s/file?id=wjVg/PLOSOne_formatting_sample_main_body.pdf and**

**https://journals.plos.org/plosone/s/file?id=ba62/PLOSOne_formatting_sample_title_authors_affiliations.pdf**

- We have carefully gone through these documents to ensure they match the style requirements.

**2. Please attach a Supplemental file of the results of the CASP quality assessment for each individual study assessed, reporting the outcome for each individual criteria considered.**

- We have reported the outcome for each of the criteria in Table 2 and have provided a modified CASP form in S3 as an example.

**3. We note that you have indicated that data from this study are available upon request. PLOS only allows data to be available upon request if there are legal or ethical restrictions on sharing data publicly. For more information on unacceptable data access restrictions, please see http://journals.plos.org/plosone/s/data-availability#loc-unacceptable-data-access-restrictions.**

**In your revised cover letter, please address the following prompts:**

**a) If there are ethical or legal restrictions on sharing a de-identified data set, please explain them in detail (e.g., data contain potentially sensitive information, data are owned by a third-party organization, etc.) and who has imposed them (e.g., an ethics committee). Please also provide contact information for a data access committee, ethics committee, or other institutional body to which data requests may be sent.**

- Our excel file of data coding was listed as ‘available from author’. There are no ethical/legal restrictions to sharing this file.

**b) If there are no restrictions, please upload the minimal anonymized data set necessary to replicate your study findings as either Supporting Information files or to a stable, public repository and provide us with the relevant URLs, DOIs, or accession numbers. For a list of acceptable repositories, please see** [**http://journals.plos.org/plosone/s/data-availability#loc-recommended-repositories**](http://journals.plos.org/plosone/s/data-availability#loc-recommended-repositories)**.**

- The excel files of coding was submitted to Dryad: <https://doi.org/10.5061/dryad.8pk0p2nqs>. Data remains unpublished during the review process and can be accessed here: <https://datadryad.org/stash/share/DSWnUl_i6XiOAmsQmehksryRzaq3JI7su69eECjBbzs>.

**We will update your Data Availability statement on your behalf to reflect the information you provide.**

**4. In your Data Availability statement, you have not specified where the minimal data set underlying the results described in your manuscript can be found. PLOS defines a study's minimal data set as the underlying data used to reach the conclusions drawn in the manuscript and any additional data required to replicate the reported study findings in their entirety. All PLOS journals require that the minimal data set be made fully available. For more information about our data policy, please see http://journals.plos.org/plosone/s/data-availability.**

**Upon re-submitting your revised manuscript, please upload your study’s minimal underlying data set as either Supporting Information files or to a stable, public repository and include the relevant URLs, DOIs, or accession numbers within your revised cover letter. For a list of acceptable repositories, please see http://journals.plos.org/plosone/s/data-availability#loc-recommended-repositories. Any potentially identifying patient information must be fully anonymized.**

**Important: If there are ethical or legal restrictions to sharing your data publicly, please explain these restrictions in detail. Please see our guidelines for more information on what we consider unacceptable restrictions to publicly sharing data: http://journals.plos.org/plosone/s/data-availability#loc-unacceptable-data-access-restrictions. Note that it is not acceptable for the authors to be the sole named individuals responsible for ensuring data access.**

**We will update your Data Availability statement to reflect the information you provide in your cover letter.**

- The minimal data set is now available in the manuscript or in dryad.

**5. Please include captions for your Supporting Information files at the end of your manuscript, and update any in-text citations to match accordingly. Please see our Supporting Information guidelines for more information: http://journals.plos.org/plosone/s/supporting-information.**

- We have included captions for the Supporting Information files, as requested.

**6. Please review your reference list to ensure that it is complete and correct. If you have cited papers that have been retracted, please include the rationale for doing so in the manuscript text, or remove these references and replace them with relevant current references. Any changes to the reference list should be mentioned in the rebuttal letter that accompanies your revised manuscript. If you need to cite a retracted article, indicate the article’s retracted status in the References list and also include a citation and full reference for the retraction notice.**

- We have not removed any references. We have added some references to respond to Reviewer #1’s request (for example, to contextualize the findings within the context of recently published reviews).

**Reviewers' comments:**

**Reviewer's Responses to Questions**

**5. Review Comments to the Author**

**Please use the space provided to explain your answers to the questions above. You may also include additional comments for the author, including concerns about dual publication, research ethics, or publication ethics. (Please upload your review as an attachment if it exceeds 20,000 characters)**

**Reviewer #1: This is a well written manuscript covering an important topic. The authors should be congratulated for their thorough and thoughtful work. I am providing some suggestions and recommendations to further strengthen the paper.**

- Thank you for your positive comments about our manuscript.

1. **Line 37 page 3. Reference 11 is noted twice in the same reference string.**

- Thank you, we have deleted this double reference.

2. **Row 43, page 3. It would be helpful to define what “child welfare processes” consist of in this context, particularly given this reviews covers a wide variety of contexts. Do these vary across country settings and how did the authors define it?**

- This is a good point to clarify. We have generally been quite broad in our understanding of child welfare (called different things by different countries, such as ‘children’s social services’ or ‘children’s aid’). We added these sentences in the Introduction to clarify: “Child welfare processes and level of service response vary by country (20). For example, most countries allow for voluntary reporting of child maltreatment (with considerable variability in mandatory reporting requirements), require reports to be investigated within a set time period, and also require that some sort of services are provided, such as services for parents (e.g., substance use treatment), for children (e.g., therapy programs), or general services (e.g., universal free medical care) (20).”
- You’ll notice from our Table 1 exclusion criteria that we focused specifically on child welfare processes and not on the types of community services to which child welfare might refer children to (e.g., mental health support). We also excluded some important child welfare processes (e.g., transitions from care) as a lot of intensive work in this area has been done, to which readers can refer. We added these sentences in the methods to clarify: “The inclusion criteria for the larger review are as follows: (1) English-language, (2) primary studies that used a qualitative design; (3) published articles; (4) investigations of children’s self-reported experiences of foster care, which could include: removal from home, foster family processes, or placement breakdown (see Table 1 for full inclusion criteria of the broader review). It could also include children’s self-reported experiences of formal and informal relationships while in foster care, including with social workers, foster carers, peers, or biological family members (siblings, parents, grandparents, aunts/uncles, cousins). The broader review excluded some themes that have been addressed by recent reviews, such as children’s self-reported experiences with violence itself (e.g., barriers to disclosure (33)), transitions out of foster care (34), and experiences at school (35).”

3. **The final paragraph in the introduction can be strengthened by adding more detail about other systematic reviews that have been conducted within this area, and how this systematic review differs/adds to that knowledge base.**

- Our review is unique in that we prioritized children’s voices in how our results were organized. This is useful for ethical reasons (children’s voices matter) and for practical reasons (to better understand and respond to children’s preferences around participation). We have clarified this in the Introduction and Discussion.

4. **Also in the final paragraph of the introduction, it seems clear that the aim is to summarize children’s perspectives. However, the reader is left to wonder, to what purpose? Who can use the information from this review and how can they use it? How is this information applicable within a practice or policy setting?**

- We hope this review will be useful for policy makers, who are increasingly requested to consider children’s voices when making decisions about their lives, and practitioners, who seem to struggle with the practicalities of how to include children’s voices. We have added this to the Introduction: “The findings of this meta-synthesis will be useful to policy makers, who are increasingly requested to incorporate children’s voices into decision-making (16,24,25), and practitioners, who need to consider the nuances of when and how to include children’s voices in practice and decision-making (26). The inclusion of children’s voices has a number of benefits to children, practitioners, and policy makers, such as affording children their inherent rights to participate, empowering children and reducing their confusion regarding services, and improving service delivery through more tailored, responsive services (24,26,27). As the authors of one review note, children’s “participation has both intrinsic (dignity and self-worth in terms of expressing views to influence decisions about their lives) and instrumental (policy and better outcomes for children in terms of supportive relationships with their workers and positive experiences at school and in CPS [children protection services]) value” (24).This meta-synthesis adds to an important increasing focus in the literature on children’s participation across all aspects of child welfare processes (24,26–28), but with an explicit focus on how foster children speak about their participation.” And this to the Discussion: “As noted in the Introduction, the findings of this meta-synthesis will be useful to both policy makers and practitioners. For example, policy makers who want to increase children’s awareness of foster care processes may find the particular areas where children expressed ‘not knowing’ of importance, such as their personal history prior to foster care; what placement or foster care means; what their foster family will be like; why they became involved with child protection services, and so on (see S5). They may also seek to improve areas of children’s experiences where they expressed considerable dissatisfaction, such as their transitions into care. Child welfare managers may want to consider areas of their practice where they are well aligned or not well aligned with children’s wishes, such as giving children information before decisions are made, changes occur or strategies to share power with children (see Table 3). They may also want to consider the qualities of professionals that children value (e.g., availability, honesty, fairness) and how these values are exemplified (or not) in their service delivery (see S6).”

5. **Search strategy, line 74. Is there a reason why key informant interviews were not included in the strategy?**

- We think that key *child* informant interviews would be included if they were available? We included index terms (“Interviews/”) and keywords (“interview*”) which should capture all types of interviews, including key informant interviews. In our experience, the inclusion of *more* search terms leads to *less* results as the results have to match the keywords (e.g., searching for “key informant interview*” would not catch “key *child* informant interviews*). But perhaps you mean other informants? Such as social workers? In which case this is a very relevant area of research that would be complementary to the findings of this review.

6. **Within the results section, there should be more information (beyond a supplementary table) regarding the CASP findings/methodological rigor of the included studies beyond inclusion of the table. What elements were weak that might have implications for the results?**

- We have made a more comprehensive table of the CASP findings so that readers can review the overall quality of the studies and for each CASP question. We have also pulled our summary of the study results from the supplementary file into the Methods section, so that there is more information about the weak/strong study elements. As noted in the Methods section, we did not exclude any studies based on methodological rigour, as we felt this would exclude important child voices. We have, however, included a note in the Discussion section that some studies were methodologically weak and that this speaks to the need for better reporting of qualitative results and continued striving to conduct ethical research with vulnerable children: “It is important to note that some studies had lower quality appraisal scores, but no studies were excluded based on quality scores so as not to exclude relevant quotes from children. Lower methodological scores highlight the need for better reporting of qualitative research and the continued need to conduct ethical research with vulnerable children.”

7. **Within the results section, findings are lumped mainly into a single table, and the discussion begins with new information that actually appears that it should belong in the results. It would be helpful to include more detailed information beyond the table within the results section, and also a more detailed analysis of findings across different contexts. For example, the participants are included from a variety of developed country settings around the world wherein the foster care system likely vastly differs. It would be helpful to provide more detailed information by either continent (or preferably) country-setting to identify if there are specific challenges/opportunities within individual contexts.**

- We gave careful consideration to how to make the results of the review accessible. Originally all of the text from Supplementary file 5 (Facets-the table and explanatory text below the table) and 6 (Facilitators and Barriers-the table and explanatory text below the table) was in the Results, which is over 4500 words of content. We have positioned this important work in the supplementary files and also have a summary table (now Table 3), so that the overall findings are more accessible to readers. We acknowledge that much of the important information is in the supplementary files and we try to draw readers attention to it in the Discussion, as you have noted. We have added more references to the supplemental files in the Discussion to strengthen the emphasis on important results; we indicate how interested readers can refer to the supplemental files for more details.
- In addition, we added the countries contributing to each theme in the right-hand column of Supplemental file 5 and 6 and added text to explain these findings in the Results (see pages 16, lines 266-276 and pages 17, lines 289-299). This provides some information about “context” to the themes. It is of note that the participation-related themes were very consistent across variable countries and contexts. However, we agree that this would be a useful area of future exploration—to better understand how contextual factors impact participation. Unfortunately, studies did not provide enough detail about the context of the foster care system in their countries to comment on this point. For example, the fact that so many studies are published from England suggests a policy shift to increase children’s voices, but this was only alluded to by the authors of one of the English studies. As the child welfare system is constantly shifting to respond to layered (often reactive) policies and procedures, we were hesitant to speculate about the context of the foster care systems in the countries at the time of data collection. We have added this important point to the limitations of the findings: “In addition, while the participation themes were remarkedly similar across the 11 high-income countries included in this review, we can hypothesize that the context of each country’s foster care system would impact the participation of children. For example, the fact that so many published studies were from England suggests a shift in policy to emphasize children’s voices, a point that was discussed by the authors of one study (42). Future research investigating the impact of participation would benefit from clear explanations of the contextual factors influencing children’s participation (e.g., details about the country’s foster care system at the time of data collection and any relevant legislation or child welfare initiatives that impact children’s participation).”

8. **In addition to the above, a more detailed understanding of how children experience the foster care system by race/ethnicity/minority status/age/gender would be very interesting.**

- Unfortunately, studies inconsistently labeled individual quotes with gender and age of the child and very rarely labeled individual quotes with other relevant factors (e.g., race/ethnicity/placement information). For this reason, we weren’t able to do this kind of in-depth analysis. This might be more possible with quantitative data. We have now included this as a limitation of our findings that deserves future attention: “As studies inconsistently labeled individual quotes (the unit of analysis) with certain demographic details (e.g., age, gender) and very rarely labeled individual quotes with other demographic information (e.g., race/ethnicity, placement information), it was not possible for us to determine how these factors influenced study findings. Future quantitative research investigating children’s participation may be able to discern if or how participation differs for children depending on their demographic factors or other information (e.g., country, child welfare context).”

**Reviewer #2: This is to me a high quality meta-synthesis of qualitative research about the experiences of foster children, a highly vulnerable population. This paper is well written. It is novel in that it appears no meta-synthesis of foster children's experiences in the qualitative literature has been done.**

- Thank you for your positive feedback. We hope the results are useful for policy makers and practitioners.
