# Supplementary material for: Foster children’s perspectives on participation in child welfare processes: A meta-synthesis of qualitative studies
Source: PLoS One. 2022 Oct 10;17(10):e0275784. doi: 10.1371/journal.pone.0275784 (PMC9550086; doi:10.1371/journal.pone.0275784)
Supplement: Attachment — Submitted filename: Responses to Editor and Reviewers_Sept 14.docx [file pone.0275784.s009.docx]

We are pleased that the Reviewers have found our revisions responsive to their comments and have submitted the updated manuscript to the Editorial Manager, as requested. Editor and Reviewer responses are found in bold below, with our responses underneath.

**Additional Editor Comments (if provided):**

**I was told that you had submitted a revised manuscript to our editorial office, and I am issuing this decision so that you can submit your revision via Editorial Manager. Hopefully a final decision can be issued swiftly after resubmission.**

- We have submitted the revision via the Editorial Manager, with the slight change to the Children Act 1989, as per Reviewer #3’s suggestion.

**Reviewers' comments:**

**Reviewer's Responses to Questions**

**Comments to the Author**

**1. If the authors have adequately addressed your comments raised in a previous round of review and you feel that this manuscript is now acceptable for publication, you may indicate that here to bypass the “Comments to the Author” section, enter your conflict of interest statement in the “Confidential to Editor” section, and submit your "Accept" recommendation.**

**Reviewer #1: All comments have been addressed**

**Reviewer #3: (No Response)**

**2. Is the manuscript technically sound, and do the data support the conclusions?**

**The manuscript must describe a technically sound piece of scientific research with data that supports the conclusions. Experiments must have been conducted rigorously, with appropriate controls, replication, and sample sizes. The conclusions must be drawn appropriately based on the data presented.**

**Reviewer #1: Yes**

**Reviewer #3: Yes**

**3. Has the statistical analysis been performed appropriately and rigorously?**

**Reviewer #1: N/A**

**Reviewer #3: Yes**

**4. Have the authors made all data underlying the findings in their manuscript fully available?**

**The PLOS Data policy requires authors to make all data underlying the findings described in their manuscript fully available without restriction, with rare exception (please refer to the Data Availability Statement in the manuscript PDF file). The data should be provided as part of the manuscript or its supporting information, or deposited to a public repository. For example, in addition to summary statistics, the data points behind means, medians and variance measures should be available. If there are restrictions on publicly sharing data—e.g. participant privacy or use of data from a third party—those must be specified.**

**Reviewer #1: Yes**

**Reviewer #3: Yes**

**5. Is the manuscript presented in an intelligible fashion and written in standard English?**

**PLOS ONE does not copyedit accepted manuscripts, so the language in submitted articles must be clear, correct, and unambiguous. Any typographical or grammatical errors should be corrected at revision, so please note any specific errors here.**

**Reviewer #1: Yes**

**Reviewer #3: Yes**

**6. Review Comments to the Author**

**Please use the space provided to explain your answers to the questions above. You may also include additional comments for the author, including concerns about dual publication, research ethics, or publication ethics. (Please upload your review as an attachment if it exceeds 20,000 characters)**

**Reviewer #1: The authors were responsive to my comments. This is a well written manuscript and is an important contribution to the literature.**

- We are pleased that Reviewer #1 found our revisions to be responsive to their helpful comments.

**Reviewer #3: I appreciate the authors’ explanation of why they were not able to address my comments previously. I also appreciate the response they have now made. As far as comment 1 is concerned, I am entirely happy. For comment 2, I would just point out that it’s the Children Act 1989 and the Children Act 2004, not the the Children’s Act, and also that the legislation only applies in England and Wales, not in the whole UK.**

- Thank you for identifying this error in our reference to the Children Act 1989. We have cited just the 1989 version, as that is what is cited by included studies. Thank you for noting the narrower range of its application (to England and Wales), which we have now specified.
